# Supplementary material for: Novel Driver Strength Index highlights important cancer genes in TCGA PanCanAtlas patients
Source: PeerJ. 2022 Aug 11;10:e13860. doi: 10.7717/peerj.13860 (PMC9375969; doi:10.7717/peerj.13860)
Supplement: Supplemental Information 2 [file peerj-10-13860-s002.zip › COHORTS/genes plots/cohorts/2021_8_16_14_9_distribution_cohorts_females_ACC.pdf]

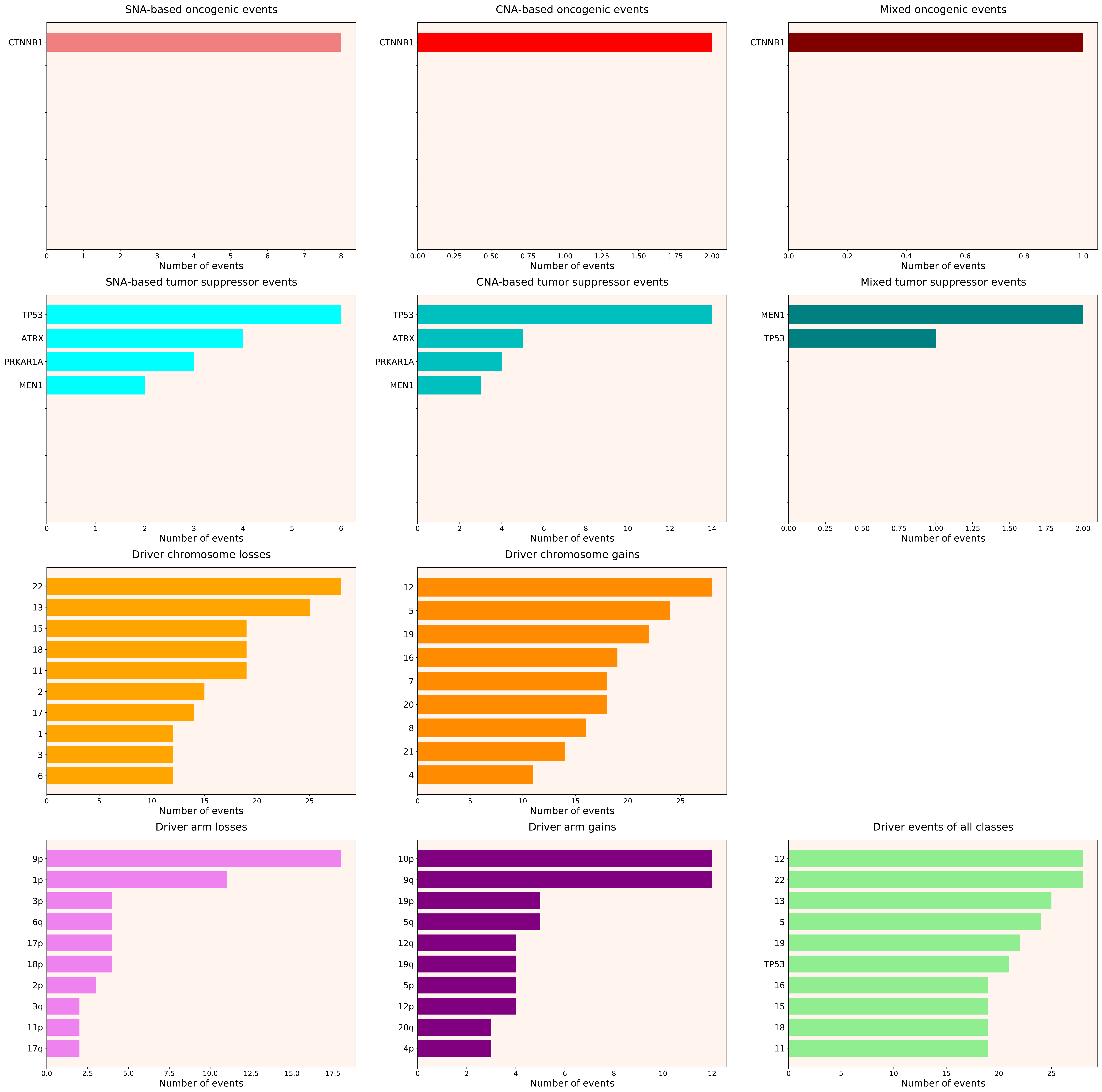

SNA-based oncogenic events
CTNNB1
Number of events
CNA-based oncogenic events
CTNNB1
Number of events
Mixed oncogenic events
CTNNB1
Number of events
SNA-based tumor suppressor events
TP53
ATRX
PRKAR1A
MEN1
Number of events
CNA-based tumor suppressor events
TP53
ATRX
PRKAR1A
MEN1
Number of events
Mixed tumor suppressor events
MEN1
TP53
Number of events
Driver chromosome losses
22
13
15
18
11
2
17
1
3
6
Number of events
Driver chromosome gains
12
5
19
16
7
20
8
21
4
Number of events
Driver arm losses
9p
1p
3p
6q
17p
18p
2p
3q
11p
17q
Number of events
Driver arm gains
10p
9q
19p
5q
12q
19q
5p
12p
20q
4p
Number of events
Driver events of all classes
12
22
13
5
19
TP53
16
15
18
11
Number of events
